# Determination of cytokine protein levels in oral secretions in patients undergoing radiotherapy for head and neck malignancies

**DOI:** 10.1186/1748-717X-7-64

**Published:** 2012-04-26

**Authors:** Deborah E Citrin, Ying J Hitchcock, Eun Joo Chung, Jonathan Frandsen, Mary Ellen Urick, William Shield, David Gaffney

**Affiliations:** 1 Radiation Oncology Branch, Center for Cancer Research, National Cancer Institute, Bethesda, MD, USA; 2 Department of Radiation Oncology, Huntsman Cancer Institute, University of Utah, Salt Lake City, UT, USA

**Keywords:** Saliva, Cytokine, Radiation, Milliplex

## Abstract

**Background:**

Cytokines may be elevated in tumor and normal tissues following irradiation. Cytokine expression in these tissues may predict for toxicity or tumor control. The purpose of this pilot study was to determine the feasibility of measuring local salivary cytokine levels using buccal sponges in patients receiving chemo-radiation for head and neck malignancies.

**Patients and methods:**

11 patients with epithelial malignancies of the head and neck were recruiting to this study. All patients received radiotherapy to the head and neck region with doses ranging between 60 – 67.5 Gy. Chemotherapy was delivered concurrently with radiation in all patients. Salivary samples were obtained from high dose and low dose regions prior to treatment and at three intervals during treatment for assessment of cytokine levels (IL-4, IL-6, IL-8, IL-10, EGF, MCP-1, TNF-α, and VEGF).

**Results:**

Cytokine levels were detectable in the salivary samples. Salivary cytokine levels of IL-4, IL-6, IL-8, EGF, MCP-1, TNF- α , and VEGF were higher in the high dose region compared to the low dose region at all time points (p < 0.05). A trend toward an increase in cytokine levels as radiation dose increased was observed for IL-6, IL-8, MCP-1, and TNF-α.

**Conclusion:**

Assessment of salivary cytokine levels may provide a novel method to follow local cytokine levels during radiotherapy and may provide a mechanism to study cytokine levels in a regional manner.

## Background

Ionizing radiation is known to increase the expression of a number of cytokines which are involved in inflammation and wound healing. These cytokines may be important in recruitment of inflammatory cells, initiation of wound repair, and development of toxicity. Cytokine levels detected in the plasma have been correlated to toxicity, such as pneumonitis [1-3]. Cytokine concentrations in tumor and circulating cytokines have also been correlated to prognosis.

Measurement of cytokines in the clinical setting typically requires plasma sampling. Plasma sampling requires consistent and rapid collection, processing, and storage techniques to minimize the risk of platelet deregulation which may falsely elevate the levels of a variety of cytokines [4-7]. Plasma levels of cytokines can also be impacted by a variety of inflammatory conditions, complicating correlations to radiotherapy in patients with inflammatory comorbidities. For these reasons, sampling plasma for research purposes can be difficult to perform and interpret in multi-institutional trials or in the community setting. A simple, non-invasive method for detecting local expression of cytokines may avoid many of these logistical and technical challenges of plasma sampling. In addition, assessment of local cytokine levels via longitudinal sampling in patients undergoing therapy may allow for an understanding of the temporal nature of cytokine elevation during radiation and correlation with tumor response and toxicity.

In the present report, we explored the feasibility of using an ophthalmic sponge (Merocel) to sample saliva and to detect cytokines in head and neck cancer patients undergoing a course of chemoradiotherapy. Ophthalmologic sponges have been used to collect secretions from human subjects for cytokine evaluation and viral recovery in prior studies [8-11]. We evaluated the ability to detect differences in cytokine concentrations in proximity to tumor (high dose region) compared to areas in the mouth planned to receive lower doses of irradiation distant to the tumor site (low dose region). Finally, we determined if salivary cytokine levels changed during the course of radiotherapy.

## Methods

### Subjects

All patients were enrolled on an IRB approved protocol at the University of Utah. Patients were eligible for this study if they were at least 18 years of age, had a Karnofsky performance score >50, were scheduled to receive fractionated radiotherapy for a pathologically documented head and neck malignancy, and provided informed consent. Patients treated with intensity modulated radiotherapy and concurrent chemotherapy were eligible. Patients in whom the target region was inaccessible to radiation were excluded. For example, pateints with hypopharyngeal tumors were enrolled only if a nodal target volume was in proximity to an anticipated sampling site.

### Simulation, planning, and treatment

All patients were simulated supine with an aquaplast mask. Spiral CT was performed at 2.5 mm slice thickness, and treatment planning was performed using Eclipse (Varian Medical Systems, Palo Alto, CA). All patients received definitive chemoradiotherapy. Treatment plans were generated with intensity modulated radiation therapy or conformal radiotherapy per standard of care depending on tumor location and characteristics. Patients were evaluated weekly during the six-week course of treatment to evaluate acute radiation toxicity and tumor response. Following therapy, patients were followed with serial clinical examinations and imaging studies to evaluate for evidence of tumor recurrence and toxicity recovery.

### Specimen collection

Specimens were obtained pretreatment and after delivery of 10, 20, and 30 Gy of radiation. All specimens were collected within 30 minutes following radiation treatment. A sterile Merocel sponge (Medtronic, Jacksonville, FL) was pre-wet with 100 uL of sterile saline before use. The high dose region for sampling was selected to be in proximity to tumor, if possible, and so that it was in a region encompassed by the 90% or higher isodose line. The site for sampling for the low dose specimen was chosen so that the dose received to the area was less than 15% of the prescription dose. The anatomic location of the site of sampling for each patient was recorded in their chart. The sponge was placed in the location of sampling without rubbing or movement for 60 seconds and immediately placed in a 50 mL conical vial. In each patient, samples were obtained inside the high dose region and at a site distant to the tumor in a low dose region, typically the buccal mucosa. Samples were obtained pretreatment and after a minimum of 10 Gy (second week), 20 Gy (third week), and 30 Gy (fourth week) of treatment. Samples were immediately frozen and stored at −80°C until shipment on dry ice. Shipped specimens were stored at −80°C until use.

### Recovery of cytokines from Merocel sponges

Sponges were thawed on ice and weighed prior to recovery. Sponge tips were separated from the handle with sterile technique and were incubated with 300 uL of ice cold extraction buffer containing 10% fetal calf serum in 0.25 M NaCl. Sponges were soaked on ice in extraction buffer for 30 minutes. Sponges and liquid were transferred to co-stat Spin-x centrifuge tubes and centrifuged at 16,000 x g for 20 minutes at 4°C. The sponge was discarded and the supernatant was utilized for further analysis.

### Evaluation of cytokine levels

Concentrations of IL-4, IL-6, IL-8, IL-10, MCP-1, TNF-α, VEGF, and EGF in saliva were analyzed with the Luminex fluorescent technique, utilizing Milliplex Magentic Beads (Millipore, Billerica, MA) according to the manufacturers’ instruction. Briefly, 25 uL of assay buffer, 25 uL of sample or standard, 25 uL of matrix solution, and 25 uL of mixed bead were added to each well of a 96 well plate and incubated overnight on an orbital shaker at 4°C. Samples and standards were plated in triplicate. After washing, 25 uL of detection antibody was added to each well and incubated for one hour at room temperature. Following incubation, 25 uL of streptavidin-phycoerythrin was added to each well and incubated on an orbital shaker for 30 minutes at room temperature. The plate was washed and 150 uL of sheath fluid was added to each well. The levels of fluorescence in each standard, quality control, and sample were detected with the Luminex® 200™ (Luminex Corporation, Austin, TX). Data was subsequently analyzed using the Bio-plex manager software (Bio-Rad Laboratories, Inc. Hercules, CA). Cytokine concentrations were obtained by interpolation of the standard curve using stepwise five-fold dilution of protein standards. Standard curves were generated for each analyte with the Bio-plex manager software and sample concentrations were calculated from the standard curve.

The sensitivity of the Milliplex assay ranged from 0.6-15 pg/ml for the cytokines studied. The intra-assay precision measured as the coefficient of variation for the studied cytokines ranged from 3.2 to 11.2% The interassay precision measured as the coefficient of variation for the studied cytokines ranged from 6.4 to 15% All samples were batched by patient and a total of two plates were required to assay all samples in duplicate.

Because the volume of sample cannot be standardized with the sponge technique, concentrations of cytokines were normalized with a previously reported dilution factor used for sponge based collection methods [9,11] with the formula dilution factor = [(x - 0.064 gm) + 0.32 gm]/(x – 0.064 gm) where x is the weight of the sponge with sample, 0.064 is the average weight of 10 dry sponges, and 0.32 gm is the weight of the 300 uL of extraction buffer added for elution. The levels of each cytokine were evaluated between local and distant sampling as well as over the course of treatment at each location.

## Results

### Patient, tumor, and treatment characteristics

Eleven patients were included in this pilot study. Tumor locations of the primary tumor in these patients were oral cavity (n = 2), oropharynx (n = 5), larynx (n = 2), and hypopharynx (n = 2). Tumor characteristics are described in detail in Table 1. The mean total dose delivered was 66.68 Gy (range: 60–67.5 Gy), and the mean dose per fraction was 2.2 Gy (Range: 2.0-2.25 Gy). The mean number of days on treatment was 44 (Range: 41–47). A seven-field technique using 6-MV photon beams was used for nine of eleven patients. Opposed lateral fields with 6-MV photon beams were used for the remaining two patients.

**Table 1:** Tumor characteristics

Site	n
Oral cavity	2
Oropharynx	5
Larynx	2
Hypopharynx	2
T-stage	
T1	1
T2	3
T3	3
T4	4
N-stage	
N0	3
N1	-
N2	7
N3	1

### Salivary cytokine levels

**Figure 1:**
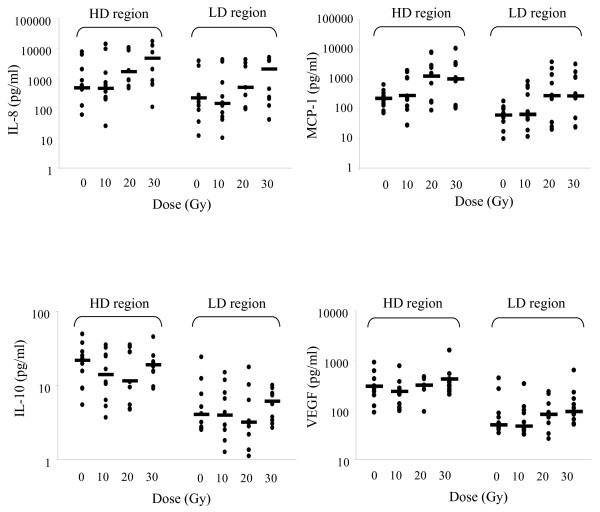
**IL-8, MCP-1, IL-10, and VEGF levels in saliva before and after irradiation.** Salivary cytokine levels were assessed with Milliplex with concentrations corrected for wet weight of the collecting sponge. Each point represents a single patient sample. HD region: within the high radiation dose region, LD region: outside of the high dose region, bar: median.

**Figure 2:**
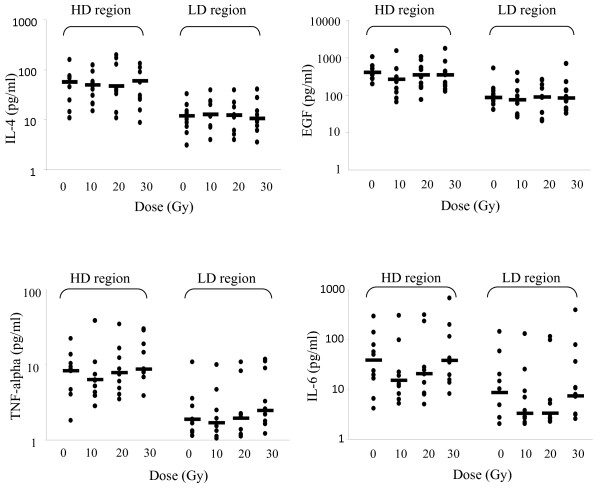
**IL-4, EGF, TNF-alpha, and IL-6 levels in saliva before and after irradiation.** Salivary cytokine levels were assessed with Milliplex with concentrations corrected for wet weight of the collecting sponge. Each point represents a single patient sample. HD region: within the high radiation dose region, LD region: outside of the high dose region, bar: median.

Salivary cytokine levels were uniformly elevated in the high dose region compared to the low dose region at baseline. Specifically, for EGF, IL-4, IL-10, MCP-1, TNF-α, and VEGF the mean baseline local levels were significantly elevated compared to baseline distant levels (p < 0.05, Table 2). Additionally, with the exception of IL-6, levels of these cytokines at the high dose region remained significantly higher than levels in the low dose region at all or most doses of radiation, suggesting that regional levels of cytokines may be detectable in saliva and may correlate to the proximity to tumor. The trends are represented graphically in Figures 1 and 2.

**Table 2:** Basal cytokine levels

	Low dose region		High dose region		Comparison (high vs low dose
Cytokine	Range (pg/mL)	Median (pg/mL)	Range (pg/mL)	Median (pg/mL)	
EGF	198.6-1037.56	346.27	40.53-508.61	86.40	p = 0.0001
IL-4	10.64-160.10	56.45	3.04-32.69	10.42	p = 0.006
IL-6	4.1-293.07	36.46	1.88-143.66	7.44	p = 0.015
IL-8	59.29-7600.26	562.36	12.1-3725.62	174.12	p = 0.032
IL-10	5.47-49.59	21.30	2.5-24.31	3.69	p = 0.0003
MCP-1	81.05-620.61	194.17	9.32-173.55	60.24	p = 0.0004
TNF-α	1.81-22.32	8.54	0.9-10.94	1.73	p = 0.0003
VEGF	89.67-881.10	278.63	34.36-431.91	51.07	p = 0.0003

### Changes in salivary cytokine levels over time

**Figure 3:**
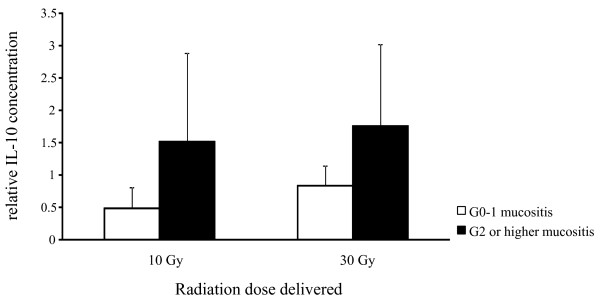
**Correlation between IL-10 levels in saliva and mucositis.** IL-10 levels were assessed with Milliplex with concentrations corrected for wet weight of the collecting sponge. IL-10 levels at 10 Gy and 30 Gy were normalized to baseline for all patients. Patients with Grade 2 or higher mucositis had a higher relative IL-10 concentration compared to those with Grade 0 or 1 mucositis. G: grade, bars: mean, error bars: SD.

As radiation dose increased, there was a trend towards increased IL-6, IL-8, MCP-1 and TNF-α levels within a sampling site (high dose region or low dose region), but this did not reach the predetermined level of significance for any analyte. In this small sample, there were no significant correlations between cytokine levels at baseline or changes in cytokine levels over time and tumor grade, tumor stage, nodal stage, local recurrence, or toxicity. However, a number of interesting non-significant trends were observed, For example, levels of IL-10, an anti-inflammatory cytokine, in saliva at each dose level compared to baseline were higher in patients with high grade mucositis compared to those with low grade mucositis (Figure 3).

## Discussion

Radiation, delivered alone or with chemotherapy, is frequently used in the definitive management of a range of malignancies, and is the primary treatment for advanced Head and Neck cancers. Due to concerns for healing during and after the delivery of radiation, assessment of tumor and normal tissue in the radiated field through biopsies or invasive techniques for correlative assays may be limited. Sampling of blood to assess changes in markers of tumor response or normal tissue toxicity may be complicated by dilution in the systemic circulation and background alterations due to other illnesses or injury. A minimally invasive technique for sampling local effects of radiation on tumor and normal tissue may provide a method to predict which patients will develop severe toxicity or allow prognostication earlier in the treatment course. Furthermore, a relatively simple, minimally invasive method for sampling could be incorporated into multi-institutional trials and simplify collection of high-quality specimens.

We have evaluated the feasibility of the collection of saliva during a course of irradiation in a pilot experiment. We were able to collect salivary samples from patients serially during the course of irradiation. These samples were not processed prior to storage and shipment, as a major goal was to develop a suitable method of sampling for multi-institutional trials in which significant processing requirements can potentially negatively impact adherence to sample collection schedules and the uniformity of sample quality. Despite the lack of additional processing at the time of sampling, we were able to successfully recover all of our target cytokines in saliva in all patients at every time point.

Buccal cell samples have been evaluated as a correlate of mucositis after radiation in a prior study [12], but this feasibility study did not evaluate a broad range of cytokines and evaluated RNA levels from buccal mucosa instead of levels of cytokines in saliva as in our study. Assessment of the protein levels in saliva is feasible in our study and changes based on location of sampling and dose delivered at the time of sampling are evident.

In several of the cytokines we analyzed, a trend toward increasing levels with increasing dose delivered was observed. It is likely that sampling at later time points may provide more impressive differences in cytokine levels in saliva compared to baseline measurements. This would likely be related to more tissue changes such as inflammation and desquamation due to higher doses of radiation. We were specifically interested in early changes, and consequently focused on the first half of definitive chemoradiation. Future studies will assess if greater differences can be observed at higher doses compared to baseline and if these differences correlate with tumor or normal tissue outcomes. One important question that will need to be answered in future studies is whether dose to salivary glands on the side of sampling correlates to changes in salivary cytokine levels. It is conceivable that the salivary gland itself may alter salivary cytokine levels to a measurable degree.

The pattern of change in traditional pro-inflammatory cytokines we evaluated may provide evidence of acute inflammation during a course of radiotherapy. For example, MCP-1 and IL-8 levels progressively increased during the course of radiation, while other cytokines, such as IL-4 and EGF were elevated in closer proximity to tumor with no trend for an increase in concentration during the course of radiation. Both MCP-1 and IL-8 result in inflammatory cell recruitment and may reflect local inflammation. A number of other possible analytes exist which could be evaluated with this technique and may correlate to acute inflammation and mucosal toxicity.

These findings raise the possibility that a technique such as salivary sampling at baseline may provide information that could be prognostic or predictive of response to therapy. Amelioration of cytokine levels in the future may be able to reduce adverse effects of radiation such as fibrosis or osteoradonecrosis. Again, additional studies in larger numbers of patients will be needed to address these questions.

## Conclusions

Sampling of salivary cytokine levels in patients undergoing radiotherapy for head and neck malignancies is feasible and may provide a non-invasive biomarker of the local effects of irradiation on tumor and normal tissue. Additional studies will be needed to clarify the potential applications and limitations of this technique.

## Abbreviations

IL, Interleukin; EGF, Epidermal growth factor; MCP-1, Monocyte chemoattractant protein −1; TNF-α, Tumor necrosis factor – alpha; VEGF, Vascular endothelial growth factor; IRB, Institutional review board; CT, Computed tomography; Gy, Gray.

## Competing interests

The authors declare that they have no competing interest.

## Authors’ contributions

DEC: Conceived of the study, assisted in design of the clinical protocol, oversaw all laboratory studies, and contributed to authorship. YJH: Assisted in study design, collection of clinical samples and data, read and contributed to authorship. EJC: Processed and analyzed specimens, read and contributed to authorship. JF: Collection of clinical samples and data, read and contributed to authorship. MEU: Processed and analyzed specimens, read and contributed to authorship. WS: Processed specimens, read and contributed to authorship DG: Conceived of the study, assisted in design of the clinical protocol, collection of clinical samples and data , and contributed to authorship. All authors read and approved the final manuscript.
